# Study protocol of a proposed Neurofeedback-Assisted Mindfulness Training Program on symptoms of anxiety and psychological distress associated with smartphone use in young adults: a randomized controlled trial

**DOI:** 10.3389/fpubh.2024.1410932

**Published:** 2024-09-04

**Authors:** Mei Fernandez-Crespo, Jose I. Recio-Rodriguez, Hsin-Chien Lee, Rosario Alonso-Dominguez, Angel L. Montejo, Laura Hernandez-Gonzalez, Virginia Iglesias Sierra, Maria I. Rihuete-Galve

**Affiliations:** ^1^Instituto de Investigación Biomédica de Salamanca (IBSAL), Salamanca, Spain; ^2^Facultad de Enfermería y Fisioterapia, Universidad de Salamanca, Unidad de Investigación de Atención Primaria de Salamanca (APISAL), Instituto de Investigación Biomédica de Salamanca (IBSAL), Red de Investigación en Cronicidad, Atención Primaria y Promoción de la Salud (RICAPPS), Salamanca, Spain; ^3^Graduate Institute of Humanities in Medicine, College of Humanities and Social Sciences, Taipei Medical University, Taipei, Taiwan; ^4^Facultad de Enfermería y Fisioterapia, Universidad de Salamanca, Instituto de Investigación Biomédica de Salamanca (IBSAL), Hospital Universitario de Salamanca, Salamanca, Spain; ^5^Unidad de Investigación de Atención Primaria de Salamanca (APISAL), Instituto de Investigación Biomédica de Salamanca (IBSAL), Gerencia de Atención Primaria de Salamanca, Salamanca, Spain

**Keywords:** mindfulness, neurofeedback, nomophobia, anxiety, young adults (18–29 years)

## Abstract

**Background:**

Nomophobia is a specific phobia characterized by the appearance of anxiety, nervousness, discomfort and distress when the mobile phone is not used and is considered an emerging public health problem because of the negative consequences on the physical and mental health of young people and adolescents, especially women. Neurofeedback-Assisted Mindfulness Training Programs may prove beneficials for improving self-control abilities, a key ability in addressing addictive behaviors. The main objective of this study is to evaluate the impact, in a young population aged 18–35 years, of an intervention based on Neurofeedback-Assisted Mindfulness Training Program (NAMTP) on disorders associated with problematic use of mobile phones. The effect of the intervention on the total score in the nomophobia test and habits of internet and social network use, as well as on signs of depression, anxiety and stress will be analyzed. As a secondary objective, the effect of the intervention on signs of insomnia will be analyzed.

**Methods and design:**

Randomized, controlled clinical-trial with two-parallel groups. 40 young adults (18–35 years) will be included and randomly assigned to Intervention Group-NAMTP or Control Group (CG). The NAMTP will include a total of 25 sessions (2-3/week) during 3-months. Each session will have a duration of 10/15 min. The instrument to be used for the neurofeedback sessions is MUSE^®^ (InteraXon Inc.). Study variables will be collected at the baseline visit and at the final visit (3-months after randomization). During these visits, questionnaires will be administered to evaluate the main and secondary variables that will include the Smartphone Addiction Scale-Short Version, Nomophobia Questionnaire, Depression, Anxiety and Stress Scale 21-item (DASS-21) and Athens Insomnia Scale.

**Discussion:**

This trial will make an important contribution to the need for evidence of effective education programs and other primary care interventions through new non-invasive interventions in reducing the risk of developing addictions to new technologies and alleviating the symptoms of discomfort associated with this problem.

**Ethics and dissemination:**

The project was approved by the Clinical Research Ethics Committee of the Salamanca Health Area (CEIm Code: PI 2023 071340).

**Clinical trial registration:**

ClinicalTrials.gov, http://www.Clinicaltrials.gov/ct2/show/NCT06188910.

## Introduction

### Problematic use of new technologies and their implications for the health of young people and adolescents

Excessive use of new technologies, including mobile phones and uninterrupted connection to the Internet, has been linked in young people to anxiety disorders, depression, attention deficit hyperactivity disorder (ADHD), personality disorders and stress, as well as to sleep problems, substance abuse, unbalanced diets, social isolation and poorer academic performance (1–3).

The excessive and problematic use of the Internet is related to syndromes such as Fear of missing out (FOMO), which is defined as the need to be continuously connected due to the “fear of missing out on something” or nomophobia which comes from the abbreviation of the English term Nomophobia (No MObile PHone PhoBIA) refers to the worry or fear that individuals experience when they are without their mobile phone or they are unable to use it. It is a specific phobia characterized by the appearance of anxiety, nervousness, discomfort and distress when the mobile phone is not used. In addition, it may precede other disorders such as depression or substance abuse (4–6).

The most susceptible and vulnerable population to suffer from nomophobia are young people and adolescents, especially women (4, 7). In addition, in the wake of the COVID-19 pandemic and lockdown, exposure to screens and use of the Internet as a means of escape and socialization has increased (8). Cases have increased to the point that excessive use of mobile phones is being considered an emerging public health problem because of the negative consequences on the physical and mental health of young people (4).

When analyzing possible factors by gender, some studies have concluded that, in women, depression has a stronger relationship with mobile phone addiction (9). The pattern of use could be behind these differences. Several studies reach similar conclusions: men use mobile phones more for leisure and entertainment (online games and video and music media applications), while women use them more for communication and socializing (social networks and applications for chatting and messaging) (7, 9).

However, lower impulse control and psychosocial and demographic factors are identified as major risk factors for nomophobia (4).

### Interventions to treat nomophobia based on neurofeedback-assisted mindfulness training programs

Currently, more and more studies are evaluating the effectiveness of different psychological, pharmacological and healthy lifestyle interventions to treat addiction to new communication technologies and the symptoms associated with their problematic use. A recent meta-analysis evaluated the effectiveness of different interventions including Cognitive-Behavioral Therapy, physical activity, pharmacological treatments or group therapy, among others. The result of this meta-analysis was that all the interventions included in the study decreased Internet addiction to a greater or lesser extent (10).

Neurofeedback (NF) is a form of biofeedback training that uses the recording of brain activity through imaging techniques to achieve, through a process of feedback, the control and regulation of brain activity patterns. Based on the principles of operant conditioning, patients learn gradually through positive reinforcement provided by feedback. Real-time information of brain activity is provided by imaging techniques such as brain magnetic resonance imaging (MRI), functional near infrared spectroscopy (fNIRs) or electroencephalogram (EEG), the latter being the most employed in NF because of lower cost and easier handling in its use (11). The electroencephalographic biofeedback (BF-EEG) or neurofeedback modality consists of placing electrodes or devices on the individual’s head that record the brain’s electrical activity and transmit it to a device. This activity is analyzed by a computer program that converts the EEG waves into visual, auditory or tactile signals that it sends to the patient so that he/she learns to work in a specific wave range, thus achieving the regulation of brain activity (12).

Recently, there has been increasing scientific interest in the effects of the mindfulness-based training programs to reduce stress, anxiety, depression, as well as in the improvement of sleep quality (13–15). Components common to these mindfulness therapies include focus and control of attention, consciousness of sensations and thoughts, and observation. The most consistent EEG findings associated with mindfulness training may be increased theta and alpha power (16). The study by Chow et al. showed a higher global full-band alpha amplitude in patients undergoing meditation training and in patients undergoing EEG-alpha neurofeedback training. In addition, the first group showed a lower upper alpha band ERD in frontal cortex 200–400 ms post-stimulus in the Stroop task, an effect that correlated with the higher alpha amplitudes demonstrated during the intervention (17). Other study suggested that neurofeedback may mediate the effect of mindfulness meditation (18).

NF is a therapy aimed at the prevention, optimization or rehabilitation of altered states of cortical activity. This technique has been shown to be effective in a variety of conditions including anxiety, depression, attention deficit disorder, post-traumatic stress syndrome (PTSD) and insomnia. It has also been used to improve cognitive performance such as memory and attention (11, 19). NF techniques have been shown to be effective in the treatment of anxiety. In 2017, clinical guidance published by the Canadian Agency for Drugs and Technologies in Health (CADTH) suggested that, compared to receiving no treatment, there was significant improvement when NF is used in patients with PTSD and Generalized Anxiety Disorders (12).

Certain training in mindfulness techniques, focused on focusing attention on the present moment, have been shown to reduce the effects of nomophobia related to problematic mobile phone use (20). Some research has shown that even a single session of mindfulness can alleviate your symptoms (18). Several studies have shown that mindfulness is beneficial for improving self-control abilities, a key ability in addressing addictive behaviors (18, 20), achieving, among other effects, an increase in alpha power (waves related to focus and attention) in the EEG study.

In view of the unstoppable development of new technologies and their increasingly widespread use in our society, there is a need to investigate the health problems derived from their inappropriate use. Interventions based on Neurofeedback-Assisted Mindfulness Training Programs could improve anxiety and other symptoms related to the problematic use of internet and new technologies.

The main objective of this study is to evaluate the impact, in a young population aged 18–35 years, of an intervention based on Neurofeedback-Assisted Mindfulness Training Program on disorders associated with problematic use of mobile phones and internet. The effect of the intervention on the total score in the nomophobia test and habits of internet and social network use, as well as on signs of depression, anxiety and stress will be analyzed. As a secondary objective, the effect of the intervention on sleep quality and signs of insomnia will be analyzed.

## Methods and analysis

### Design

The design of this study corresponds to a clinical trial with two parallel randomized groups.

### Study population

The study participants will be recruited in the consultations of the urban Health Centers of the Salamanca Health Area. Subjects will be included by consecutive sampling taking into account the following selection criteria:

Inclusion criteria: age between 18 and 35 years, having one or more Smartphone-type mobile devices, having a compatible mobile phone to install and use the MUSE^®^: EEG Meditation & Sleep application (the description of this device and its operation is developed later in this manuscript), being fully functional (physically and mentally free of known disorders and disabilities), willing to voluntarily participate in the study and signing the informed consent form.

Exclusion criteria: History of severe psychiatric disorders (such as schizoaffective disorders, bipolar disorder, major depressive episode with symptoms or other non-organic psychotic disorders) requiring psychiatric treatment in the 6 months prior to inclusion in the study, history of brain injury or other problems contraindicating the use of neurofeedback.

### Sample size calculation

The sample size estimation is based on the expected results in one of the main variables, which is the change in anxiety symptoms. This estimate was made using the results collected in the study by Abdian et al. (21), who conducted a study similar to the one we propose using the DASS-21 anxiety scale score as a change variable, obtaining a mean difference between groups after the intervention of 3.9 points in this questionnaire. Accepting an alpha risk of 0.05 and a beta risk of 0.2 in a bilateral contrast, 40 participants (20 participants in the experimental/intervention group and 20 in the control group) are required to detect a clinically relevant difference equal to or greater than 3.9 points on the DASS-21 anxiety questionnaire. The statistical power of a hypothesis test is 93% to detect the difference between the means as statistically significant. The common standard deviation on this questionnaire, according to data provided by Abdian et al. (21) is assumed to be 3.7. A loss to follow-up rate of 30% has been estimated.

### Inclusion, randomization and procedures

**Figure 1:**
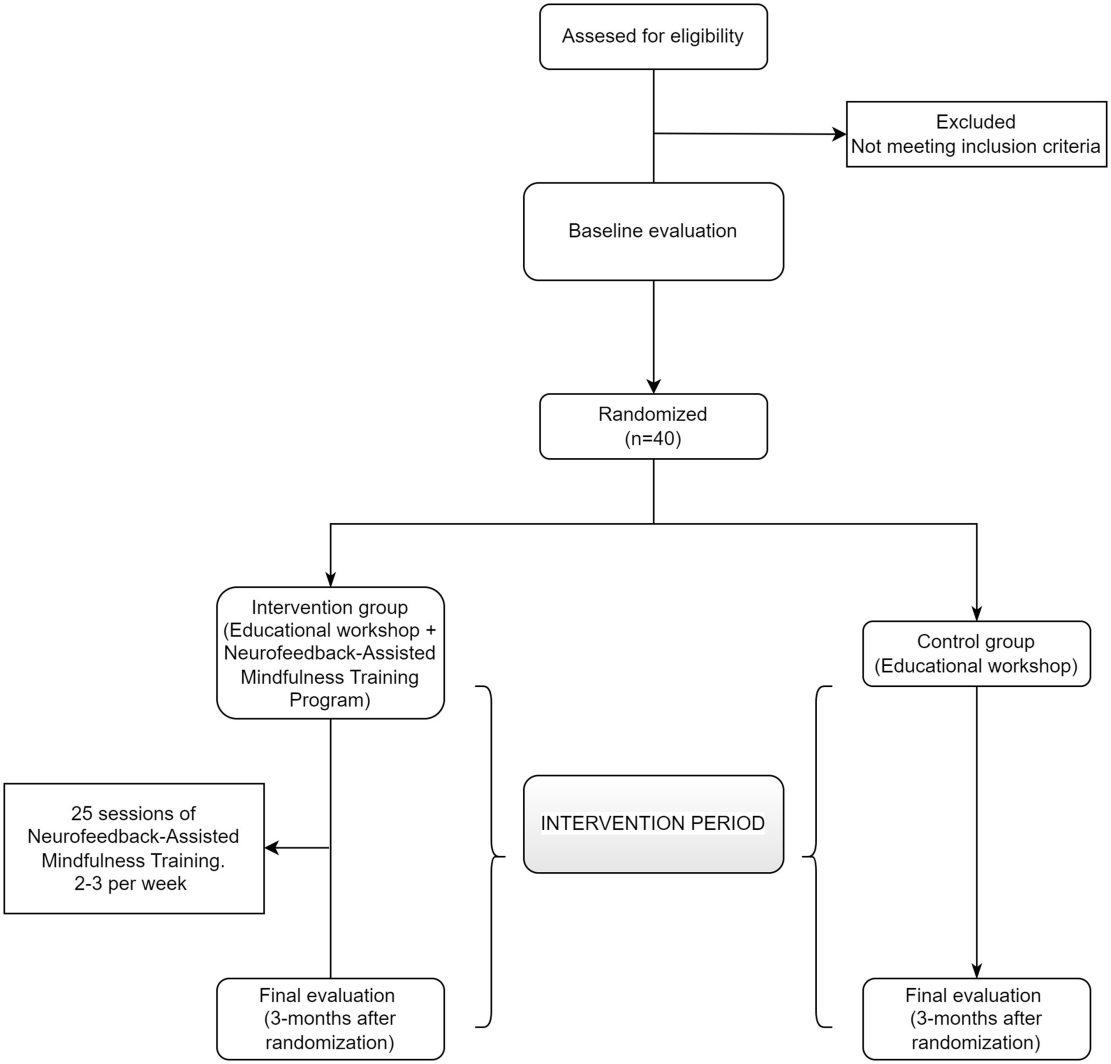
Flow-chart diagram of the study.

A researcher will contact by telephone to arrange a visit with the selected individuals at the health centers. During this visit, the study will be presented, any doubts will be resolved and the informed consent form will be signed. After verifying that the subject meets the inclusion criteria, a baseline evaluation will be carried out in which clinical and sociodemographic variables and the study change variables will be collected. Finally, each participant will be randomly assigned to one of the 2 groups (Intervention group (IG)- Neurofeedback-Assisted Mindfulness Training Program) or Control Group (CG). Randomization will be performed randomly using the Epidat 4.2 program (22) with a ratio of 1/1. The allocation will be kept hidden until participants are assigned to each group. A final evaluation will be performed on all participants at the end of the study (3 months after randomization) to re-evaluate clinical, sociodemographic and study change variables (Figure 1).

The study, evaluations and intervention will be carried out at the University Hospital of Salamanca and the Institute of Biomedical Research of Salamanca (IBSAL). The recruitment of participants will be carried out in the primary care clinics of the Salamanca Health Area, Spain.

### Study variables

Study variables will be collected at the baseline visit and at the final visit (3 months after randomization).

Primary outcomes:

- Smartphone addiction: The short version of the Smartphone Addiction Scale (SAS-SV), validated in Spanish by Lopez-Fernandez (23), will be used. This scale consists of 10 items and scores higher than 31 are considered susceptible to the presence of addiction to mobile phones (23). In addition, the age at the beginning of the use of new technologies, age of the first mobile phone, type of devices used to connect to the Internet, hours of daily use of the mobile phone, most frequent activities carried out with the mobile phone (calls and messages, games, entertainment, social networks, Internet search, etc.) will also be collected.
- Nomophobia: The Nomophobia Questionnaire (NMP-Q) will be used for its evaluation. It is a questionnaire composed of 20 items and validated in Spanish by González-Cabrera (24). The range of scores varies from 20 to 140 points. Scores above 20 would indicate the presence of nomophobia (24).
- Depression, anxiety and stress: The Spanish version of the Depression, Anxiety and Stress Scale 21-item (DASS-21) was used to assess the presence of negative emotional states (25). This questionnaire consists of 21 items divided into three subscales or categories of 7 items each: depression, anxiety and stress. Each item is scored according to the presence of symptoms during the last week on a four-point Likert scale. The final score is obtained with the sum of the scores for each subscale. The DASS-21 has been used to assess nonclinical samples in different age ranges, including adolescence (26). The presence of adverse mental health symptoms was considered according to the cut-off points proposed by Roman et al. (27) in the validation study with a sample of young people in Spain: DASS-21-Depression≥6 points, DASS-21-Anxiety≥6 points, and DASS-21-Stress≥5 points.

Secondary outcomes:

- Sleep quality and signs of insomnia: It will be evaluated through the Athens Insomnia Scale (AIS). This is a tool that helps in the diagnosis and assessment of insomnia according to ICD-10 diagnostic criteria. It consists of 8 items, with four response options scoring from 0 to 3. A higher score on the scale indicates the presence of insomnia symptoms (28).

Other variables:

- In the baseline evaluation, age, sex, educational level, and employment status will be collected. The level of physical activity will be asked by administering the International Physical Activity Questionnaire (IPAQ). For this purpose, the short 7-item version validated in Spanish will be used. This questionnaire evaluates the intensity, frequency and duration of physical exercise performed in the last 7 days. It allows us to classify the level of activity as high, moderate or low (29).

### Definition of the intervention

#### Educational workshop

The educational workshop will be received by both IG and CG participants. In both cases, after randomization. This workshop called “Digital Balance: Disconnect to (re)connect” will last approximately 20 min and will address the following contents:

1. Digital balance: definition, difference between necessary time and time for recreational use in front of the screens.
2. Reflecting on the relationship with technology and new devices.
3. Establish limits: define periods of disconnection and limit the use of social networks.
4. Be aware of the content consumed on networks.
5. Identity and digital footprint on the Internet.

#### Neurofeedback-Assisted Mindfulness Training Program (NAMTP)

The NATMP will be carried out by the IG alone for a period of 3 months after randomization. A total of 25 sessions will be carried out at a frequency of 2–3 per week. Each session will have a duration of 10/15 min. The organization of the sessions is described in Table 1 of this study protocol.

**Table 1:** Description of the Neurofeedback-Assisted Mindfulness Training Program (NAMTP).

Week	Number of session	Name of the session	Program of the session	Duration
01	01	Mind meditation: A focused attention practice. Your first mind meditation	Discover Mind Biofeedback	05:19
		Intro: Muse breath meditation. Your first breath meditation	Discover Heart, Breath and Body	06:00
01	02	Anchoring attention	Discover Mind Biofeedback	07:08
01	03	Counting breaths	Discover Mind Biofeedback	07:18
02	04	Letting thoughts pass	Discover Mind Biofeedback	06:42
		Fresh Air: 4 s Box Breath	Breath	05:00
02	05	Extending the exhale	Discover Heart, Breath and Body	08:00
03	06	Posture and body positioning	Discover Mind Biofeedback	09:19
03	07	Building the habit	Discover Mind Biofeedback	11:57
04	08	Training a puppy	Muse Essentials	10:00
04	09	Sensation of breath	Muse Essentials	10:00
05	10	Counting breaths	Muse Essentials	10:00
05	11	Sitting comfortably	Muse Essentials	10:00
06	12	Finding your soundscape	Muse Essentials	10:00
06	13	Dealing with distraction	Muse Essentials	10:00
07	14	Working with discomfort	Muse Essentials	10:00
07	15	Lowering the bar	Muse Essentials	10:00
08	16	Finding clarity and playfulness	Muse Essentials	10:00
08	17	Mind Biofeedback meditation (EEG) Unguided	Mind	10–15 min
09	18	Mind Biofeedback meditation (EEG) Unguided	Mind	10–15 min
09	19	Mind Biofeedback meditation (EEG) Unguided	Mind	10–15 min
10	20	Mind Biofeedback meditation (EEG) Unguided	Mind	10–15 min
10	21	Mind Biofeedback meditation (EEG) Unguided	Mind	10–15 min
11	22	Mind Biofeedback meditation (EEG) Unguided	Mind	10–15 min
11	23	Mind Biofeedback meditation (EEG) Unguided	Mind	10–15 min
12	24	Mind Biofeedback meditation (EEG) Unguided	Mind	10–15 min
12	25	Mind Biofeedback meditation (EEG) Unguided	Mind	10–15 min

**Figure 2:**
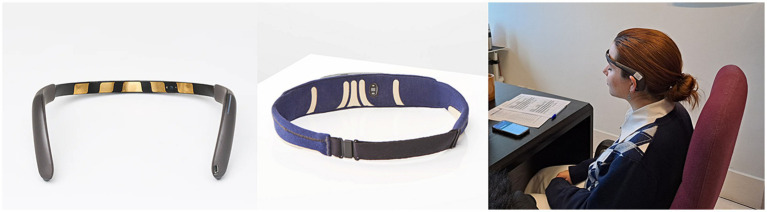
MUSE^®^ 2 and MUSE^®^ S Headbands (InteraXon Inc.).

The instrument to be used for the neurofeedback sessions is MUSE^®^ (InteraXon Inc.), using two models MUSE^®^ 2 Headband and MUSE^®^ S Headband. MUSE^®^ is a multi-sensor headband device that has been tested and validated against EEG systems and provides real-time information on brain activity, pulse and respiration. Figure 2 shows the MUSE^®^ headbands. These headbands use 7 finely calibrated sensors—2 on the forehead, 2 behind the ears plus 3 reference sensors. MUSE^®^ is a state-of-the-art EEG system that uses advanced algorithms to train beginner and intermediate meditators to control their concentration. It teaches users to manipulate their brain states and change the characteristics of their brain. Unlike traditional neurofeedback (which focuses on training individual frequencies), the MUSE^®^ app goes further and incorporates a unique and complex combination of various brainwaves to deliver results such as calm, active and neutral states.

Both models, MUSE^®^ 2 Headband and MUSE^®^ S Headband, record EEG signals at a sampling rate of 256 Hz, which are transferred via a Bluetooth connection with the MUSE^®^: EEG Meditation & Sleep mobile app. The MUSE^®^ app transforms and processes brain signals using Machine Learning techniques into information that it returns to the individual for personalized NF-based training. Read more: https://choosemuse.com/.

MUSE^®^ devices and the MUSE^®^: EEG Meditation & Sleep application have been used in clinical studies analyzing anxiety (30) and stress (31), as well as in interventions to manage obsessive compulsive disorders (32) or in the assessment of brain performance (33).

At the beginning of each session, participants completed an initial calibration process in which the device determined the user’s baseline EEG profile over a period of time involving concentration versus distraction. The main proprietary measure of “calm” produced by MUSE^®^ has a sampling frequency of 1 Hz. It is a measure of momentary attention to breathing, with a range of 0–100. MUSE^®^ also produces a measure of “recoveries” for each session, i.e., the number of times the user refocuses on calm breathing after an episode of mental wandering. A third measure, “birds,” represents moments of sustained attention for longer than 5 s. At the end of each session, a registration form should be completed following the model shown in Table 2.

**Table 2:** Record form for each session.

Number of the session:	Name of the session:		Type of session: □ Guided □ Non-Guided
Data:		Duration:	
MIND			
% Calm:	Active mind time:	Neutral mind time:	Calm mind time:
STILLNESS			
% Still:	Active time:		Relaxed time:
HEART RATE			
Average Heart rate:	High Heart rate:		Low Heart rate:
BREATHING			
% High harmony:	Time low harmony:	Time media harmony:	Time high harmony:
No recoveries:		No birds:	
*How did you feel during the session?* 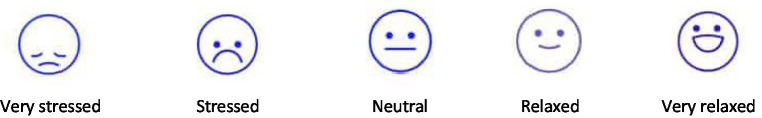			

### Data analysis

Variables will be collected in a database in the SPSS v.28 statistical package. An intention-to-treat analysis will be performed, so that the results of all individuals entering the study will be analyzed, regardless of whether or not they complete the study protocol. This will allow us to assess the effect according to the percentage of adherence to the sessions, as well as to avoid biased calculations by including all participants. The normality of the variables collected will be evaluated using the Kolmogorov–Smirnov test (34). The characteristics of the sample will be expressed by frequency distribution and percentages for categorical variables; and calculation of mean and standard deviation for quantitative variables. In the case of parametric distributions, the association between variables will be analyzed with Student’s *t*-test and analysis of variance (ANOVA). In the case of not following a normal distribution, the corresponding nonparametric tests of the previously mentioned tests will be used. The Student’s *t*-test (Mann Whitney U) for independent samples will be used to compare the means between two groups, evaluating the change within the same group with the Student’s *t*-test for paired data (Wilcoxon test). The relationship between quantitative variables will be analyzed using Pearson’s correlation coefficient or Spearman’s correlation coefficient for non-normal variables. A repeated measures analysis (a between-groups repeated measures ANOVA) will be performed as well as an estimation of the parameters and the effect size. For the bilateral contrasts of hypotheses an alpha risk of 0.05 is set as the limit of statistical significance.

## Discussion

The relevance of the study in terms of its clinical, healthcare and/or technological development impact: This work includes some of the policy priorities of the Europe 2020 strategy that address major concerns shared by citizens such as understanding the determinants of health, using an interdisciplinary approach that integrates biological, epidemiological, environmental, socioeconomic and behavioral approaches. In relation to the clinical impact, the results of this work could be used to design improved interventions to change behaviors, as well as education programs and other primary care activities through new non-invasive methodologies that are effective in reducing the risk of developing addictions to new technologies and alleviating the symptoms of discomfort associated with this problem. In relation to technological development, this project will serve to evaluate the clinical use of MUSE^®^ neurofeedback devices and to create specific intervention protocols to address anxiety or addiction problems.

The relevance of the project in terms of scientific and academic impact: The group’s intention is to publish the results in international journals of different specialties. In addition, the study is part of a Doctoral Thesis by compendium of articles. Finally, it is intended to inform society by broadcasting to the media, in addition to small multimedia videos and podcasts with the main results for wide dissemination in social networks.

### Limitations

The study follows all the recommendations of the CONSORT guidelines, but due to the nature of the intervention, the participating subjects will not be blinded to the intervention. However, both the evaluators and the investigators responsible for data analysis will be blinded. Maximum adherence to the intervention will be attempted with reminders with date and time of the next session.

## Ethics and dissemination

This study has been approved by the Clinical Research Ethics Committee of the Salamanca Health Area (CEIm Code: PI 2023 071340) and was carried out in accordance with the Declaration of Helsinki. Participants were informed of the objectives of the study and signed an Informed Consent form. The confidentiality of the participants’ data was guaranteed at all times in accordance with the provisions of the Organic Law 3/2018, of December 5, on the Protection of Personal Data and guarantee of digital rights and Regulation (EU) 2016/679 of the European Parliament and of the Council of 27 April 2016 on the protection of individuals with regard to the processing of personal data and the free movement of data (GDPR).
